# Impact of pulsed electric fields and mechanical compressions on the permeability and structure of *Chlamydomonas reinhardtii* cells

**DOI:** 10.1038/s41598-020-59404-6

**Published:** 2020-02-14

**Authors:** Sakina Bensalem, Dominique Pareau, Bertrand Cinquin, Olivier Français, Bruno Le Pioufle, Filipa Lopes

**Affiliations:** 1 Université Paris-Saclay, ENS Paris-Saclay, CNRS, Institut d’Alembert, SATIE, Gif-sur-Yvette, 91190 France; 2 Université Paris-Saclay, CentraleSupélec, LGPM, Gif-sur-Yvette, 91190 France; 3 Université Paris-Saclay, ENS Paris-Saclay, CNRS, Institut d’Alembert, LBPA, Gif-sur-Yvette, 91190 France; 4 ESIEE-Paris, ESYCOM Université Paris-Est, Noisy-le-Grand, 93160 France; 5 Université Paris-Saclay, ENS Paris-Saclay, CNRS, Institut d’Alembert, LuMIn, Gif-sur-Yvette, 91190 France

**Keywords:** Biotechnology, Electrical and electronic engineering

## Abstract

Current research findings clearly reveal the role of the microalga’s cell wall as a key obstacle to an efficient and optimal compound extraction. Such extraction process is therefore closely related to the microalga species used. Effects of electrical or mechanical constraints on *C. reinhardtii*’s structure and particularly its cell wall and membrane, is therefore investigated in this paper using a combination of microscopic tools. Membrane pores with a radius between 0.77 and 1.59 nm were determined for both reversible (5 kV∙cm^−1^) and irreversible (7 kV∙cm^−1^) electroporation with a 5 µs pulse duration. Irreversible electroporation with longer pulses (10 µs) lead to the entry of large molecules (at least 5.11 nm). Additionally, for the first time, the effect of pulsed electric fields on the cell wall was observed. The combined electrical and mechanical treatment showed a significant impact on the cell wall structure as observed under Transmission Electron Microscopy. This treatment permits the penetration of larger molecules (at least 5.11 nm) within the cell, shown by tracking the penetration of dextran molecules. For the first time, the size of pores on the cell membrane and the structural changes on the microalgae cell wall induced by electrical and mechanical treatments is reported.

## Introduction

Microalgae are currently recognized as a potential renewable source of proteins, pigments and lipids with a broad range of industrial applications (cosmetics, food and feed, chemical, …)^1^. When cultured under nitrogen starvation, some microalgae accumulate large amounts of neutral lipids in the form of droplets in the cytoplasm. They are therefore considered as a promising renewable source of energy and a potential alternative to traditional fossil fuels^2,3^.

However, several scientific, technological and economical bottlenecks must be overcome before microalgae become the primary source for large-scale production of biodiesel. The main steps that need optimization to become less costly economically and environmentally are the cultivation, harvesting and extraction step^1,4–6^.

In the particular case of extraction, this usually includes the use of organic solvents, highly toxic to human health and the environment^7,8^. A pretreatment consisting of cell disruption before placing microalgae in contact with a solvent is commonly used in order to improve extraction^9,10^. This pretreatment is of utmost importance as the quantity and quality of the compounds obtained from the microalgae are very dependent on the treatments used. In addition, the chosen treatment must take into account the microalgal strain used. Currently, both steps of cell disruption and lipid extraction remain highly expensive^11^.

Several studies involving the use of cell disruption methods prior to solvent extraction were published in the past few years. It is widely accepted today that the major obstacle to an optimal extraction is the cell wall of microalgae whose properties are highly dependent on the species and on the environment in which they grow. Indeed, several microalgal species (*Chlorella sp*., *Nannochloropsis sp*. and *Haematococcus sp*….), known to have a significant potential as renewable sources for biofuel and other bio compounds, are surrounded by a thick, sometimes multi-layered, cell wall that needs to be disrupted before proceeding with the extraction step^10,12^. Specifically, in the case of lipid extraction, it has been demonstrated that the solvent’s extraction efficiency is enhanced when the cell wall is weakened^13–16^.

A thorough comprehension of the microalga’s cell structure and in particular, the cell wall, is therefore highly required in the research of an optimal extraction process including the most adequate pretreatment. Comprehensive studies of the microalgae’s cell wall structure in the context of an extraction process using pretreatments are however, currently poorly investigated.

The cell disruption process is either non-mechanical, such as physical (e.g. microwaves and pulsed electric fields), chemical (supercritical CO_2_) and biological (enzymatic degradation) or mechanical such as bead milling or high-pressure homogenization^9,17^. Combinations of pretreatments, such as sonication and homogenization, have also shown promising results for an improved lipid extraction^18^. In addition, an innovative method of electroporation through PEF (Pulse Electric Fields) application combined to organic solvents has proven to be simple, fast and highly efficient as a pretreatment to extract compounds from microalgae as intracellular lipids^19–22^.

This work focuses on electrical (PEF) and mechanical pretreatments. Their impact on the cell structure and morphology of *C. reinhardtii* was studied. This is mandatory when aiming at developing an efficient pretreatment process.

*C. reinhardtii* is a well-known microalga that has been studied for many years in biological research^23,24^ and most recently as the study model for the production of bio products^25–27^. *C. reinhardtii* is a unicellular, biflagellate green alga, with a 7-layered cell wall structure, composed of hydroxyproline-rich glycoproteins. This structure intrigued many scientists and was a subject of debate due to divergences of opinion on the composition of each layer^28–30^. In the case of the optimization of compound extraction from *C. reinhardtii*, it is significantly important to take into consideration this cell wall structural complexity.

Therefore, in this study, the effect of PEFs, mechanical compressions and the combination of both pretreatments on *C. reinhardtii*’s morphology and structure (cell wall, cell membrane and inner components) was assessed using a combination of microscopic tools (Transmission Electron Microscopy (TEM) and Confocal Laser Scanning Microscopy (CLSM)). Furthermore, the size of pores on the cell membrane after submitting the microalgae to the different pretreatments was determined through an innovative method combining CLSM acquisition and image analysis using fluorescent labelled molecules (FITC-Dextrans) of different sizes.

## Methods

### Microalgae strain

This study was held on the green, unicellular microalga *Chlamydomonas reinhardtii* (SAG 34.89) acquired from the Culture Collection of Algae at the University of Göttingen, Germany (SAG).

### Microalgae cultivation

The algae strains were cultured in 250 mL flasks.

#### Growth conditions

For the growth of the microalgae strain, the following conditions were applied: a constant temperature of 25 ± 0.5 °C, continuous agitation at 100 rpm, atmospheric CO_2_ (0.04%), and mean light intensity of 20 μmol·m^−2^·s^−1^. The algae was cultivated in TAP medium (Gorman and Levine, 1965) for an optimal growth (with a conductivity of 0.184 ± 0.001 S·m^−1^ and a pH around 7), at a concentration ranging from 2 × 10^5^ to 1 × 10^7^ cells·mL^−1^ and in a total volume of 50 mL. The culture medium conductivity evolved and was 0.089 ± 0.001 S·m^−1^ (pH ≈ 8.55) after 4 days of growth conditions.

#### Stress conditions

After growing in the previous conditions, the cells were centrifuged at a rate of 6000 g for 5 min. To induce lipid accumulation, they were cultured with the same agitation and temperature as above and under stress conditions: nitrogen depleted TAP medium (TAP N-: removal of NH_4_Cl, conductivity of 0.119 ± 0.001 S·m^−1^ and pH around 7) and higher light intensity (150 μmol·m^−2^·s^−1^). The culture medium conductivity in this case, and after 7 days of stress conditions, was of 0.081 ± 0.001 S·m^−1^ (pH ≈ 8.8). The cells from the growth phase were resuspended in TAP N- medium (total volume of 60 mL) at a concentration of 3 × 10^6^ cells·mL^−1^. For all experiments, the cell sample was concentrated to a final value of 6 × 10^6^ cells·mL^−1^ before treatment.

### Pretreatments

#### Pulsed electric field (PEF) treatment

*Chlamydomonas reinhardtii* cells were submitted to a PEF treatment using a bipolar pulse generator (Betatech Electrocell B10 HVLV, potential range of −1000 V to +1000 V), in plastic electroporation cuvettes (Bio-Rad) with an electrode distance of 1 mm. The cell samples, with a total volume of 130 µL, were electroporated using monopolar pulses at two pulse durations of 5 and 10 µs and potentials of 550 or 700 V, depending on the applied electric field. For all PEF treatments, the following parameters were kept constant: pulse repetition frequency of 100 ms and a burst of 10 monopolar pulses. The PEF parameters leading to reversible and irreversible electroporation were determined previously on *C. reinhardtii* cells^31^ by monitoring the penetration of the Sytox Green (0.6 kDa) in the cells using flow cytometry. Under those conditions, a pulse duration of 5 µs with an electric field intensity of 5 kV∙cm^−1^ leads to the formation of reversible pores while 7 kV∙cm^−1^ creates irreversible pores.

Moreover, in our previous study^31^, we demonstrated that membrane pores resealed in less than 1 minute in cells submitted to reversible conditions. We therefore consider here that membrane pores of cells submitted to irreversible conditions remain open when we observe the sample under the confocal microscope (20 min after PEF exposure, see further below in this Methods section).

#### A combination of PEFs with mechanical compressions

In the case of this pretreatment, PEFs were combined with a cyclic mechanical compression of the cells through microfluidic constrictions. The detailed description of the microfluidic system is included in our previous work^15^.

**Figure 1:**
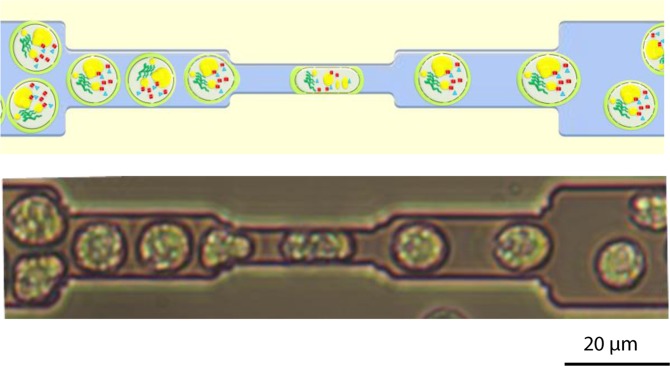
Mechanical compression of *C. reinhardtii* cells in the microfluidic system. *Upper image*: schematic drawing of the mechanical compression process; the electroporated cells, enriched in compounds of interest (lipids, protein, pigments…), are compressed by flowing through the microfluidic restrictions (height/width: 5 µm) leading to a higher permeabilisation of the cell. *Lower image*: observation of the mechanical compression of *C. reinhardtii* cells under bright field microscopy.

In brief, soft lithography standard protocol^32^ was used to fabricate the microfluidic device. The design includes 8 analysis frames containing 24 parallel micro channels, each composed of 10 restrictions. The cells, that are about 10 µm in diameter, flow through the restrictions included in these micro channels as it is shown in Fig. 1. The dimension of each restriction, generating a compression on the cell, is 5 µm in width and in height. The cells were forced through the restrictions using a microfluidic pressure pump at a pressure of 1000 mbar and therefore with a flow rate of 1.11 µL·s^−1^.

The algal cells were first submitted to a PEF treatment (5.5 kV·cm^−1^, 5 µs) before flowing through the microfluidic constrictions for a cyclic mechanical compression (10 compressions per cell). The resulting cell sample was then analysed for microscopic observation and pore size characterization. In addition, to further assess the impact of mechanical compressions on the penetration of bigger dextran molecules (40 kDa and 70 kDa), cells were submitted to a PEF treatment followed by a higher number of constrictions (two-fold increase in the number of constrictions). The time delay before analysis was similar for both experiments.

### Pore size characterization

The characterization of the pore size induced by the different pretreatments was held through an innovative method combining Confocal Laser Scanning Microscope (CLSM, Confocal Leica SP8) image acquisition and analysis (see hereafter). Accordingly, fluorescent dye (Concanavalin A) was used to detect the cell wall and FITC-Dextran molecules of different sizes were used for estimating the cell’s permeability. Cells were first incubated with the dextran molecules then submitted to the different pretreatments and then finally stained by Concanavalin A.

#### FITC-dextrans

In this study, various fluorescein-labeled (Excitation/Emission (nm): 488/495–540) dextran molecules were tested; they differ by their molecular weight: 3, 10, 40 and 70 kDa. All dextran molecules were purchased from Thermo Fisher Scientific (Invitrogen).

Aqueous solutions of the 4 different dextran molecules were prepared in an aqueous buffer with a final concentration that depends on their maximal solubility. The latter decreases as the molecular weight increases. The final concentration for each dextran was therefore 500 µM for 3 kDa dextran, 500 µM for 10 kDa dextran, 250 µM for 40 kDa dextran, and 100 µM for 70 kDa dextran.

The dextran solution was then added to the cell sample (volume: 100 µL; cell concentration in the range of 6 × 10^6^ – 1 × 10^7^ cells·mL^−1^) to obtain a final concentration of 50 µM for 3 kDa dextran, 37.5 µM for 10 kDa dextran, 10 µM for 40 kDa dextran and 2.5 µM for 70 kDa dextran. The final dextran concentrations were chosen in respect with the sample volumes used in our experiments. The ratio of dextran molecules per cell is from 1.5∙10^8^ to 3∙10^9^. The incubation time before confocal microscopy analysis was 20 min; this value was chosen after a short kinetic study performed between 10 and 30 min.

Dextrans are hydrophilic polysaccharides, composed of glucose molecules that are linked by α-1,6 glycosidic linkages. These glucose polymers can be defined by their equivalent coil radius that was calculated by the method described by Flory (1953) (see hereafter).

To determine the size of a polymer, different properties must be taken into consideration such as its structure, its composition and the way it behaves in the chosen fluid. In our work, they are dissolved in a liquid solution (solvent) where they naturally form random coils thanks to the flexibility of the polymer’s backbone. The size of a polymer should therefore correspond to the size of the coil that is formed. To simplify the calculations, linear polymers with chemically identical monomers are chosen as the model for our study.

P. J. Flory (1953) considered a polymer as a long and ideal polymeric chain (which represents a simple and first approximate model for a specific polymer chain). This means that it is composed of monomers with very few interactions between them and that are bonded to each other by highly flexible linkages. In the case of a “good solvent”, in which the polymer is soluble, as water for hydrophilic polymers, the polymer chain is much expanded and its interactions with the solvent molecules favoured. In contrast, in a “bad solvent”, the polymer is less soluble and the chain stays very condensed as the monomer-monomer interactions are favoured over the monomer-solvent interactions. The radius will therefore depend on the nature of the solvent in which the molecule is diluted. Flory (1953) defined a radius of gyration or coil radius (R_g_) for the polymer molecule as:

1 $${R}_{g}=\frac{{R}_{0}}{\sqrt{6}}$$

where ${R}_{0}=a{N}^{\nu }$, with a, the monomer size, N, the degree of polymerization (number of bond segments), and ν, the Flory exponent. For a good solvent, ν is equal to 3/5. Considering the fact that dextrans are polymers composed of many glucose molecules^33^, the monomer size here is estimated at approximately 0.35 nm (hydrodynamic radius of a glucose).

In our case, the dextran molecules were diluted in DMSO which is considered a good solvent and after in water (a good solvent for hydrophilic molecules).

An equivalent coil radius was then calculated for each dextran molecule (Table 1).

**Table 1:** Determination of the equivalent coil radius for each dextran molecule^34^.

Dextran MW (Da)	R_g_ (nm) *Coil radius*	N *Degree of polymerization*
3000	0.77	16.7
10000	1.59	55.5
40000	3.66	222
70000	5.11	388.6

#### Cell wall staining

The lectin Concanavalin A (ConA), conjugated with tetramethylrhodamine, was used to visualize *C. reinhardtii*’s cell wall as it binds to α-mannopyranosyl and α-glucopyranosyl residues (C860, Molecular Probes, Inc., Invitrogen). When bound to its targets, the tetramethylrhodamine conjugate emits an orange-red fluorescence (Excitation/Emission (nm): 561/571–620). A stock solution was prepared by dissolving the reagent in sodium bicarbonate (0.1 M, pH ≈ 8.3), at a final concentration of 2 mg∙mL^−1^. Finally, 20 µL of the ConA stock solution was added to the cell sample and incubated during 20 min before microscopic observation.

#### Analytical method

Different fluorescein-labeled dextran molecules were used to determine the minimal size of pores formed on the algae’s cell membrane after the different pretreatments.

A specific method was developed to automatically evaluate the size of the pores created in the membrane. The first step consisted of acquiring confocal microscopy images of the microalgal cells right after they have been submitted to the pretreatment and stained with ConA for the observation of their cell wall. Then two additional images were acquired: one corresponding to chlorophyll autofluorescence (Excitation/Emission (nm): 405/550–650) and the other on light transmission. These two last images were mostly used for quality control and for testing the remaining integrity of the cells.

**Figure 2:**
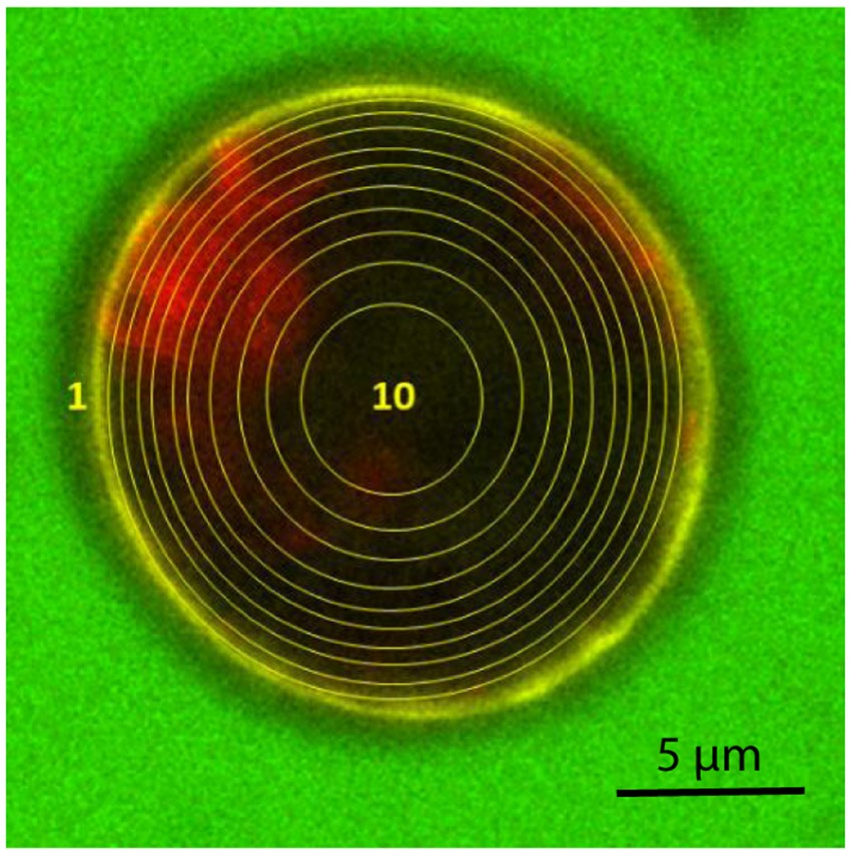
CLSM image of *C. reinhardtii* after a PEF treatment (5.5 kV/cm, 5 µs, 10 pulses). The cell is electroporated in a medium containing 3 kDa Dextran-FITC. It is segmented into nine concentric rings: from ring n°1, that excludes the cell wall, to ring n°9) and one inner circle (n°10), all equal in terms of area. Scale bar: 5 µm. The chlorophyll is detected in red, the cell wall is stained in yellow by ConA and dextran is detected in green (the green fluorescence inside the cell isn’t easily visible but can be further analysed as shown in the section Methods)^34^.

This method was written in ImageJ Macro language, option Bio-Formats (see Supplementary Fig. S1). It can be described as follows: an automatic segmentation of the microalgal cell image is performed using the “cell wall” channel (ConA) to determine the limit between the extracellular and intracellular media. A mask is then created and applied to the dextran channel to determine the presence or absence of dextran fluorescence inside the cell. The cells, chosen of approximately equal diameters, are each automatically divided into 10 zones of equal area: nine concentric rings (from ring 1, corresponding to the first zone of the cell after the cell wall and membrane, to ring 9) and one inner circle (n°10) (as shown in Fig. 2). The segmentation of the cell into several zones is necessary to compare the fluorescence intensities in the different parts of the cell. Finally, the mean dextran fluorescence intensity emitted by each zone is measured. The segmentation of the cell into several zones of same area is necessary to compare the fluorescence intensities in the different parts of the cell. We then normalize this fluorescence intensity calculating its ratio over the average mean fluorescence intensity of the background (surrounding medium).

Exclusion size filters were also used to sort out cell debris or exceptional individuals (gigantic microalgae, multibodies…). From the individual intensity profiles acquired for about 10 to 20 microalgal cells, an average profile and standard deviations can be deduced for each applied conditions.

### *C. reinhardtii* – cell structure and morphology

The impact of PEF treatment and mechanical compressions on *C. reinhardtii*’s cell structure and morphology was monitored using a JEOL JEM-1400 Transmission Electron Microscope (TEM) operating at 120 kV. Images were acquired using a postcolumn high-resolution (11 megapixels) high-speed camera (SC1000 Orius; Gatan) and processed with Digital Micrograph (Gatan) and ImageJ. To preserve the state of the algal cells during analysis, the medium solution was previously removed by centrifugation (6000 g for 5 min) and the cells were incubated over night at 4 °C with 4% glutaraldehyde in 0.1 M cacodylate buffer at pH 6.8. The cells were then washed with cacodylate 0.1 M, pH 6.8, post-fixed 1 h at room temperature in 1% osmium and 1,5% potassium hexacyanoferrate in cacodylate 0.1 M and washed in water. Cells were then embedded in small cubes of 2% LMP-agarose to facilitate their manipulation and dehydrated in ethanol series (10–30 min in ethanol 10%, 20%, 30%, 40%, 50%, 70%, 90%, 2 times in 100%) and propylene oxide (30 min). Embedding in resin was performed over 2 days, with increasing concentration of resin mixed with propylene oxide (resp. 25%, 50%, 75% and pure resin), before polymerisation 24 h at 60 °C.

The solid algal samples were then cut into semithin (250 nm) or ultrathin sections (80 nm) with an ultramicrotome EM UC6 (Leica Microsystems) and respectively collected on glass slides or on Formvar carbon-coated copper grids (Agar Scientific) before observation. Semithin sections were stained with methyleneblue-azur II and observed with a Leica DM750 microscope for the screening of the blocks. Ultra-thin sections were stained with 2% uranyl acetate (Merck) and lead citrate (Agar Scientific).

## Results and Discussion

*C. reinhardtii* cells were submitted to three different pretreatments: PEFs, a combination of PEFs with cyclic mechanical compressions and a combination of PEFs with an increased mechanical compression (by increasing the number of successive compressions applied on the algal cell).

The impact of PEF parameters on cell morphology and structure was tested for two electric field intensities, 5.5 kV∙cm^−1^ and 7 kV∙cm^−1^ (respectively corresponding to reversible and irreversible electroporation^31^), with a pulse duration of 5 µs. The combined pretreatments included this first step of PEF, followed by mechanical compressions where the cells are flowed within successive microfluidic constrictions of 5 µm (width and height). The control corresponds to 7-day stressed cells that were not submitted to any pretreatment.

The impact of these pretreatments was evaluated by investigating i) the cell’s morphology and structure (chloroplasts, cell membrane and wall) and ii) by estimating the size of the pores created on the cell membrane through a combination of complementary microscopic tools and image analysis.

### Cell structure: impact of pretreatments

**Figure 3:**
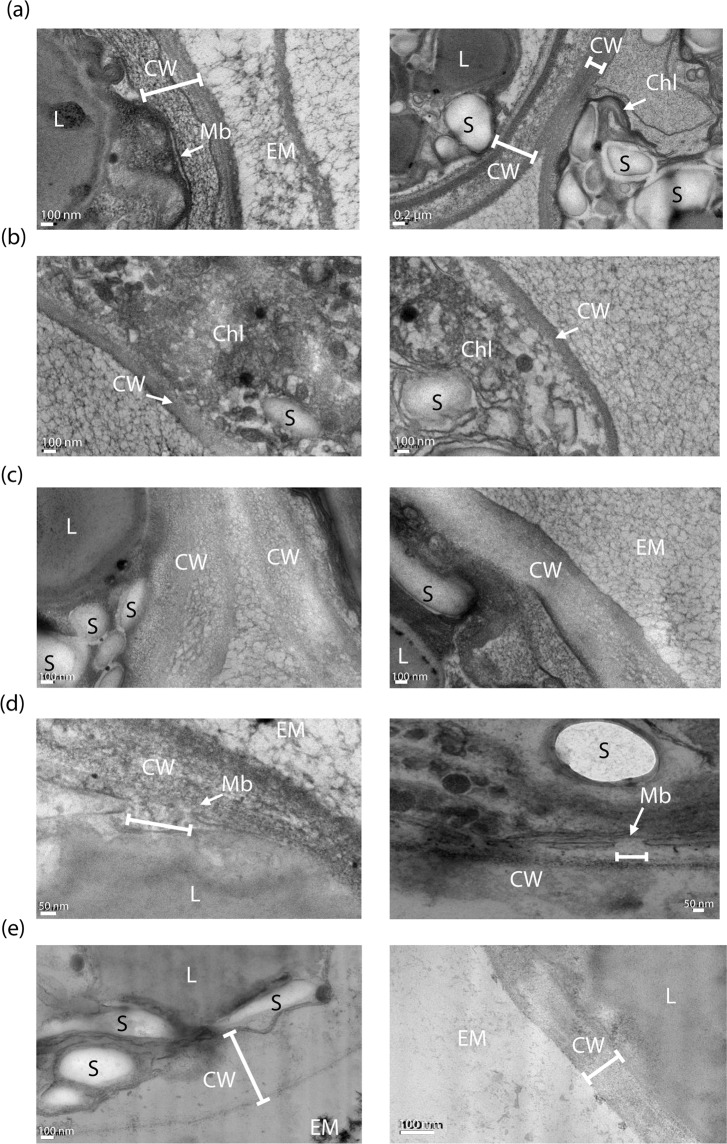
TEM observations showing the impact of PEF and mechanical compressions on 7-day stressed *C. reinhardtii* cells. **(a)** Control; the integrity of the membrane and cell wall (left image) and chloroplast (right image) can be observed, **(b)** cells submitted to reversible electroporation (5.5 kV∙cm^−1^, 5 µs); both images show the structural impact on the chloroplasts, **(c)** cells submitted to reversible (left image) and irreversible (right image) electroporation (7 kV∙cm^−1^, 5 µs), **(d)** cells submitted to irreversible electroporation; a pore on the membrane can be seen, **(e)** cells submitted to the pretreatment combining reversible electroporation and mechanical compressions. **CW**: Cell wall; **L**: lipid droplets; **S**: Starch; **Chl**: Chloroplast; **Mb**: Plasma membrane; **EM**: Extracellular Medium^34^.

*C. reinhardtii* cells were characterized by TEM. Figure 3 demonstrates a clear impact of the pretreatments, PEF and mechanical compression, on the chloroplasts, the cell’s membrane and the multi-layered cell wall.

First, these microscopic observations show the impact of electroporation on the chloroplast. Compared to the control (Fig. 3a (right image), 7-day stressed cells that weren’t submitted to any pretreatment), the grana structure appears much less organized after the pretreatment involving reversible electroporation (Fig. 3b). The impact of irreversible electroporation (Fig. 3d) on the cell membrane, where pores were created after the treatment, is also detected. It should be stressed that the pore’s size inferred from the image cannot be compared to that determined by the analytical method we developed (see section hereafter and the Methods section) since the preparation of the sample before observation is different.

As previously reported, *C. reinhardtii’s* cell wall presents a multi-layered structure^28,29,35^. These observations are consistent with our previous data obtained with confocal laser scanning microscopy^34^. Images show that in the case of both reversible and irreversible electroporation, the cell wall is less structured (Fig. 3c) compared to that of the control (Fig. 3a). Its complex structure did not appear as clearly as it was in the case of the control (Fig. 3a). Besides, when the cell is pretreated by the combination of both PEF and mechanical compressions, the cell wall seems even less structured, sometimes inexistent or uneasy to perceive as the different layers and limits between the cell membrane and cell wall are very hard to distinguish (Fig. 3e). Overall, the PEF treatment influenced both the cell membrane and wall, while the mechanical compressions impact mainly the cell wall^15^. In addition, we show, for the first time, an effect of PEF on the microalgae cell wall.

### Cell membrane: pore size characterization

#### Impact of PEF pretreatment

The size of the pores created on the membrane, after the cell was submitted to different pretreatments, was determined according to the method described before (see section Methods), using various sizes of fluorescein-labelled molecules: 3 kDa, 10 kDa, 40 kDa and 70 kDa dextrans.

**Figure 4:**
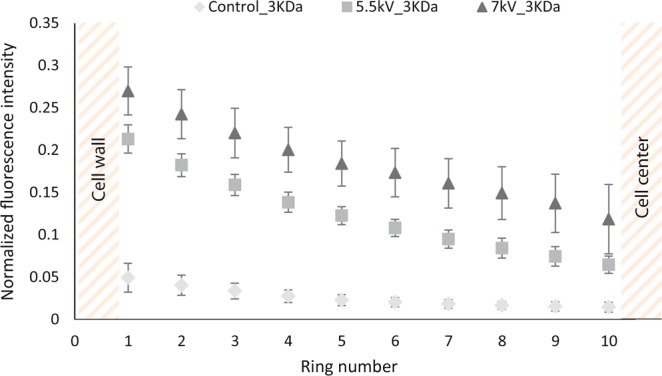
Fluorescence intensity emitted by 3 kDa dextran in algal cells previously submitted to reversible electroporation (5.5 kV∙cm^−1^) and irreversible electroporation (7 kV∙cm^−1^); ratio of the fluorescence intensity of 3 kDa dextran emitted in each zone by the fluorescence intensity emitted in the medium. The other PEF parameters were kept constant: burst of 10 pulses, repetition frequency of 10 Hz and pulse duration of 5 µs. The control corresponds to 7-day stressed cells that weren’t submitted to any pretreatment^34^.

Figure 4 shows the mean fluorescence intensity of the 3 kDa dextran in the 10 zones of the cell that are defined in the section Methods. In the case of the control, the mean fluorescence intensity is minor and negligible. The control therefore shows no entry of the 3 kDa in the cell. On the other hand, dextran penetration within the cell is clearly observed for both reversible (5.5 kV∙cm^−1^) and irreversible (7 kV∙cm^−1^) PEF conditions, with a pulse duration of 5 µs and with a similar spatial distribution.

For both PEF treatments, a significant decrease of the mean fluorescence intensity of the dextran from the outer ring to the centre of the cell is observed. The distribution of the molecule inside the cell is then inhomogeneous, which could be explained by the rather slow diffusion of dextran from the wall to the centre of the cell; the presence of inner components, especially lipid droplets, in the cytoplasm could decrease the dextran’s diffusion. Indeed, after 7 days of stress conditions the algal cells used in these experiments were highly rich in lipid droplets^15,36–38^.

In addition, these results indicate that the minimal pore radius, created in these PEF conditions, is 0.77 nm (Table 1). To determine the maximum pore size, 10, 40 and 70 kDa dextran molecules were tested in the same reversible and irreversible conditions. The experiments showed no entry for the 10 kDa dextran in reversible conditions (where pores reseal in less than 1 min). We therefore consider that bigger dextran molecules are not able to enter the cell under these reversible conditions either. Under irreversible conditions, all sizes were tested. No entry was observed for any of those larger dextran molecules (10 kDa, 40 kDa nor 70 kDa; see Supplementary Fig. S2). In conclusion, pores with a radius between 0.77 and 1.59 nm (corresponding to the 10 kDa dextran) are estimated for both reversible and irreversible PEF conditions with a pulse duration of 5 µs (see Table 1).

**Figure 5:**
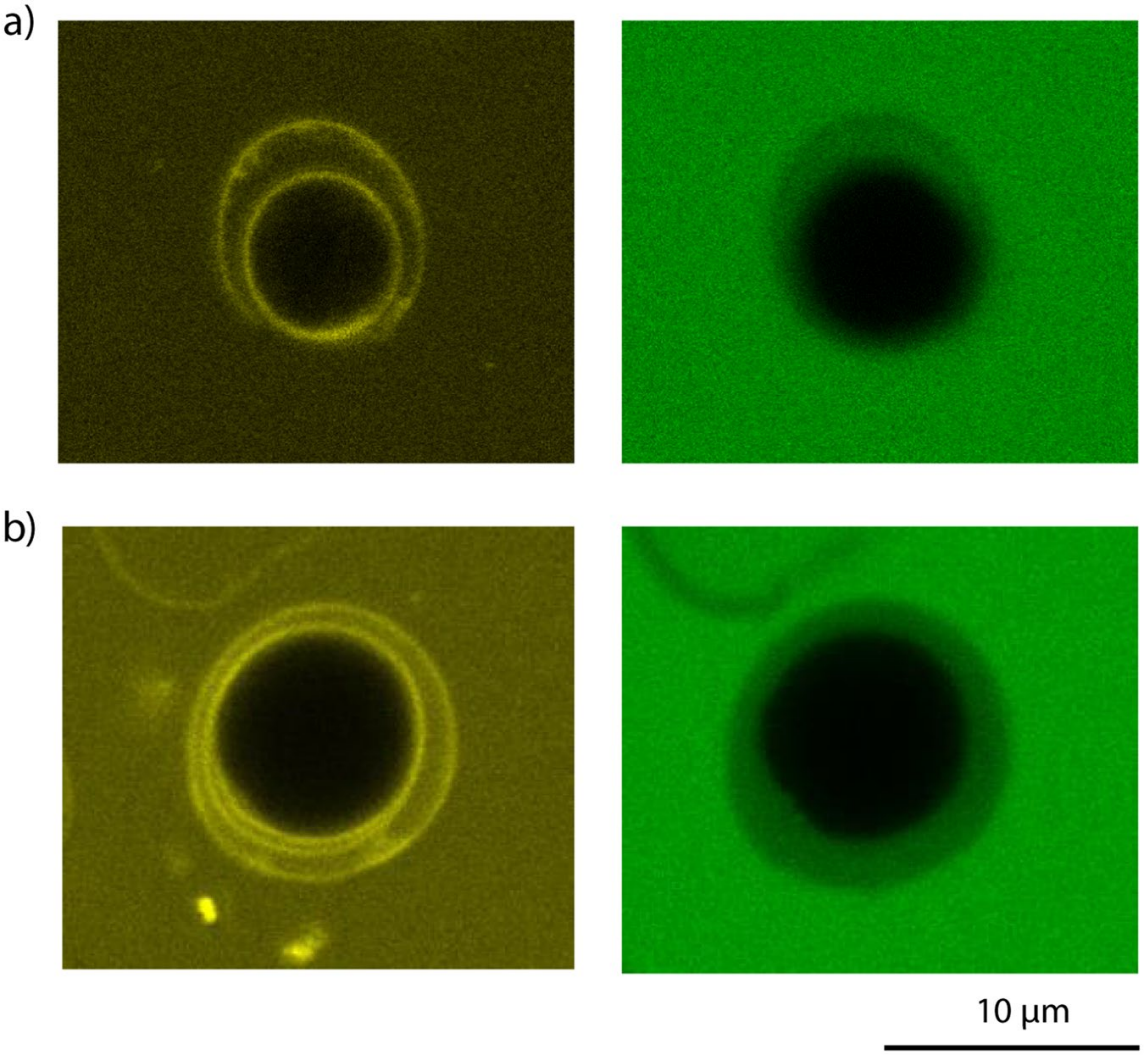
Confocal microscopy images for the analysis of the penetration of the 3 kDa dextran molecule in the cell wall of *C. reinhardtii* under stress and growth conditions. *Left image*: visualization of the cell wall stained with Con A (yellow fluorescence). *Right image*: visualization of the green fluorescence emitted by the dextran molecule in the cell wall. **(a)** 7 day-stressed cells; **(b)** cells under growth conditions^34^.

Interestingly, the microscopic observations acquired during these experiments showed that the 3 kDa dextran could penetrate the cell wall of 7-day stressed *C. reinhardtii* not submitted to any pretreatment. On the other hand, despite the penetration of the 3 kDa dextran molecule in the cell wall as shown in Fig. 5a, the molecule did not pass through the membrane; no dextran fluorescence was detected in the cytoplasm (see Fig. 4).

On the contrary, the larger dextran molecules (10 kDa, 40 kDa and 70 kDa) did not show any penetration in the cell wall (see Supplementary Fig. S3). The cell wall of the 7-day stressed algal cells is therefore naturally permeable to molecules of a radius between 0.77 nm (corresponding to the 3 kDa dextran) and 1.59 nm (corresponding to the 10 kDa dextran) while the cell membrane seems to constitute the main barrier to the entrance of the molecule of 3 kDa size. Interestingly, this was also observed in the case of cells under growth conditions (Fig. 5).

Furthermore, the effect of longer pulses was tested. We found that 10 µs pulses of 7 kV∙cm^−1^ could let penetrate dextran molecules up to 70 kDa (see Supplementary Fig. S4). Consequently, the expected minimal pore radius is of 5.11 nm in these conditions.

This result is consistent with previous publications, showing that higher pulse durations lead to larger pores on the membrane of different types of cells^39,40^. In addition, this result also suggests a possible effect of longer pulses on the cell wall that is now permeable to the bigger dextran molecules. This is also consistent with the TEM observations in Fig. 3. This is an important observation that should lead to deeper investigation on the effect of longer pulses on not only the cell membrane but also the cell wall.

#### Impact of PEF with mechanical compressions pretreatments

**Figure 6:**
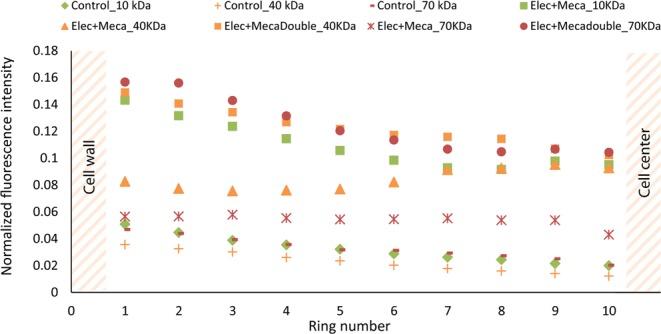
Impact of pretreatments on *C. reinhardtii*’s permeability to different sizes of dextran-FITC molecules (5.5 kV∙cm^−1^, 5 µs); ratio of the fluorescence intensities of zones 1 to 10 by the fluorescence intensity emitted in the medium. The control corresponds to 7-day stressed cells that were not submitted to any pretreatment, “Elec” to an electroporation under the following conditions: 10 unipolar pulses, 5.5 kV∙cm^−1^, 5 µs pulse duration and a repetition frequency of 10 Hz, “Elec + Meca” to the combination of the electroporation with mechanical compressions (10 per cell) and “Elec + Mecadouble” to the same combination of pretreatments with a higher amount of mechanical compressions (20 per cell)^34^.

Secondly, the impact of the combination of PEFs with mechanical compressions was investigated in this work. Results are shown in Fig. 6, where the normalized intensity of each zone from ring 1 to circle 10 (vs control) is plotted.

For this experiment, the electric field intensity was equal to 5.5 kV∙cm^−1^ and the pulse duration to 5 µs, therefore corresponding to reversible conditions. As previously stated, the reversible electroporation only led to the penetration of the 3 kDa dextran.

The results in Fig. 6 clearly show that cells submitted to these PEF conditions combined with a mechanical solicitation are now permeable to the 10 kDa dextran. Under these conditions, the mechanical compression either enlarged the pores from a radius compatible with the entry of the 3 kDa dextran (0.77 nm) to a radius compatible with the entry of 10 kDa dextran (1.59 nm) and/or affected the cell wall structure/permeability allowing the entry of the latter molecule in the cells. This is consistent with TEM observations (see Fig. 3e). Indeed, the cell wall structure is impacted by the combined pretreatments.

**Figure 7:**
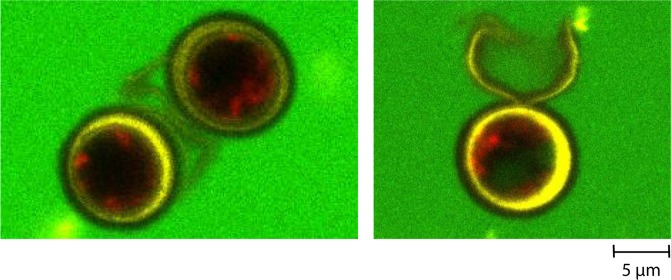
Observation under confocal microscopy of the 40 kDa dextran penetration in 7-day stressed *Chlamydomonas reinhardtii* cells before (left image) and after being pretreated with a combination of PEF and mechanical compressions (right image: visualization of the dextran 40 kDa inside the algal cell). The cell wall is stained with ConA (yellow fluorescence), the auto-fluorescence of the chlorophyll is detected in red and the fluorescence of the dextran molecule in green^34^.

In the case of the 40 kDa and, in minor extent for 70 kDa dextran, an increase in mean fluorescence intensity of the cells pretreated with a combination of PEF and mechanical compression was measured (Fig. 6) and observed under confocal microscopy (see Fig. 7, right image). Interestingly, when the cells were submitted to a combination of PEF and a higher number of mechanical compressions (two-fold), an increase of the fluorescence intensity was observed for both the 40 kDa and the 70 kDa dextran: they then significantly penetrated the cell in those conditions in contrast with the previous pretreatment.

This result highlights the impact of mechanical compressions on the permeability of 7-day stressed *C. reinhardtii* to the dextran molecules. An increase in the number of compressions applied on the algal cell and therefore a longer mechanical stress duration, leads to the higher uptake of various sizes of dextrans. This might be explained by the enlargement of the pores from 1.59 nm to at least 3.66 nm and/or to an increase in permeability due to a less structured cell wall.

## Conclusions

This work investigated the effect of the pretreatments PEF and mechanical compressions on the structure and morphological properties of *C. reinhardtii*. For the first time a clear impact of both pretreatments was assessed on the cell wall; this was confirmed by TEM observations that showed a much less dense and structured cell wall.

In addition, membrane pore size was estimated for two PEF conditions by monitoring the penetration of dextran molecules of various sizes. Pores with radius within 0.77 to 1.59 nm were detected for short pulses (5 µs) while larger irreversible pores (at least a radius of 5.11 nm) were obtained with 10 µs-long pulses (7 kV·cm^−1^).

In all cases *C. reinhardtii’s* cell wall showed permeability towards the 3 kDa dextran molecule.

When cells were submitted to additional mechanical constraints, the penetration of bigger dextran molecules was detected. This might be attributed to both the enlargement of the membrane pores and/or a higher cell wall permeability due to a less structured cell wall.

Overall, these results are significant when aiming at developing a selective extraction of compounds from microalgae. Indeed, tuning the intensity of the mechanical forces and the PEF conditions (electric field amplitude and pulse duration) might lead to the possible size-dependent extraction of molecules of interest from microalgae. Such perspectives will therefore be investigated in our future work.

## Supplementary information

Supplementary information

## Data Availability

The data that support the findings of this study are available from the corresponding author upon reasonable request.
