# Supplementary material for: Impact of pulsed electric fields and mechanical compressions on the permeability and structure of Chlamydomonas reinhardtii cells
Source: Sci Rep. 2020 Feb 14;10:2668. doi: 10.1038/s41598-020-59404-6 (PMC7021699; doi:10.1038/s41598-020-59404-6)
Supplement: Supplementary file 1 — Supplementary information [file 41598_2020_59404_MOESM1_ESM.pdf]

Impact of pulsed electric fields and mechanical compressions on the permeability and structure of *Chlamydomonas reinhardtii* cells

Sakina Bensalem[1,2,*], Dominique Pareau[2], Bertrand Cinquin[3], Olivier Français[4], Bruno Le Pioufle[1], Filipa Lopes[2]

[1]ENS Paris-Saclay, CNRS, SATIE UMR 8029, Université Paris-Saclay, Cachan 94230, France

[2]CentraleSupélec, Université Paris-Saclay, LGPM EA 4038, Gif-sur-Yvette 91190, France

[3]ENS Paris-Saclay, CNRS, LBPA UMR 8113, Université Paris-Saclay, Cachan 94230, France

[4]ESIEE-Paris, ESYCOM, Université Paris-Est, Noisy-le-Grand 93160, France

* Corresponding author - Sakina Bensalem at : SATIE, UMR CNRS 8029, Ecole Normale Supérieure Paris Saclay, 61 av du Pdt Wilson 94230 Cachan, France

E-mail address: sakina.bm@gmail.com (Sakina Bensalem)

```
//Macro "Intensity Measurements into micro-algae"
//Code written by Bertrand Cinquin : bertrand.cinquin@espci.fr using ImageJ V1.52p
//free of use
run("Set Measurements...", "area mean area_fraction redirect=None decimal=3");
//Draw the contour of the algae you wish to investigate
run("Measure");
area1= getResult("Area",0);
Radius = sqrt(area1/(3.1416));
print("Area is", area1, "Radius is", Radius);

        roiManager("Add"); //Add the outter ring of the algae
        for(i=1;i<10;i++){ //Define 10 rings in a way that each area defined between each ring are equal
                roiManager("Select",0);
                print(i,(10-i)/10,sqrt((10-i)/10)*Radius);
                Diff=Radius*(1-sqrt((10-i)/10));
                run("Enlarge...", "enlarge=-"+Diff);
                roiManager("Add");
    }
        for(i=1;i<10;i++){ //Define the areas
                l = i-1;
                Selec =newArray(l,i);
        roiManager("Select",Selec);
                roiManager("XOR");
        roiManager("Add");
        }
        for(i=10;i<19;i++){  //Measure the different areas
        roiManager("Select",i);
                run("Measure");
        }
        roiManager("Select",9);   // The last ring is also the last area and therefore measured last
        run("Measure");
```

Supplementary Figure S1 Analytical method used in this study written in Image J Macro language; available at: https://github.com/BCinquin/Micro-Algae/blob/master/Macro_MicroAlgae.ijm

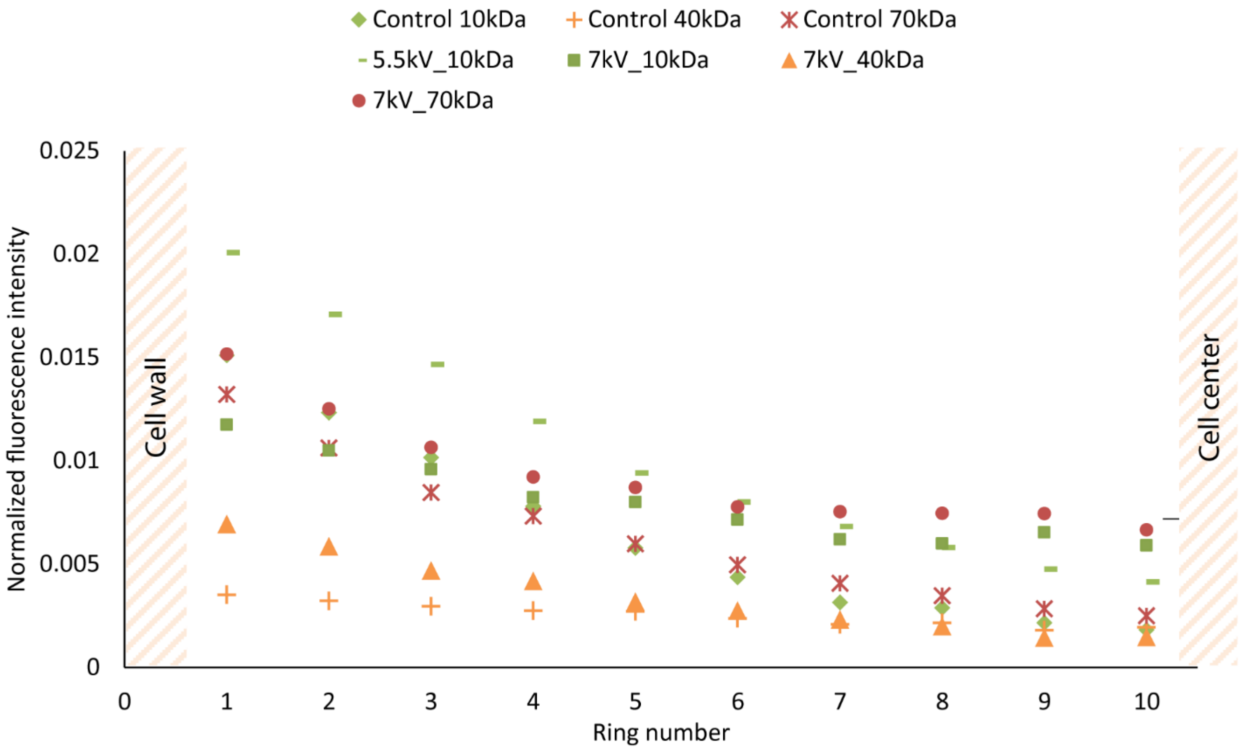

Supplementary Figure S2 Fluorescence intensity emitted by various dextran molecules (10 kDa, 40 kDa and 70 kDa) in algal cells previously submitted to reversible electroporation (5.5 $kV \cdot cm^{-1}$) or irreversible electroporation (7 $kV \cdot cm^{-1}$); ratio of the fluorescence intensity of given dextran emitted in each zone by the fluorescence intensity emitted in the medium. These fluorescence intensities, in the range of 0.002 to 0.05 (corresponding to the intensities measured for the control samples of all experiments), are considered negligible and related to the background. Therefore, they indicate no entry of these dextran molecules inside the cell. The other PEF parameters were kept constant: burst of 10 pulses, repetition frequency of 10 Hz and pulse duration of 5 µs. The control corresponds to 7-day stressed cells not submitted to any pretreatment.

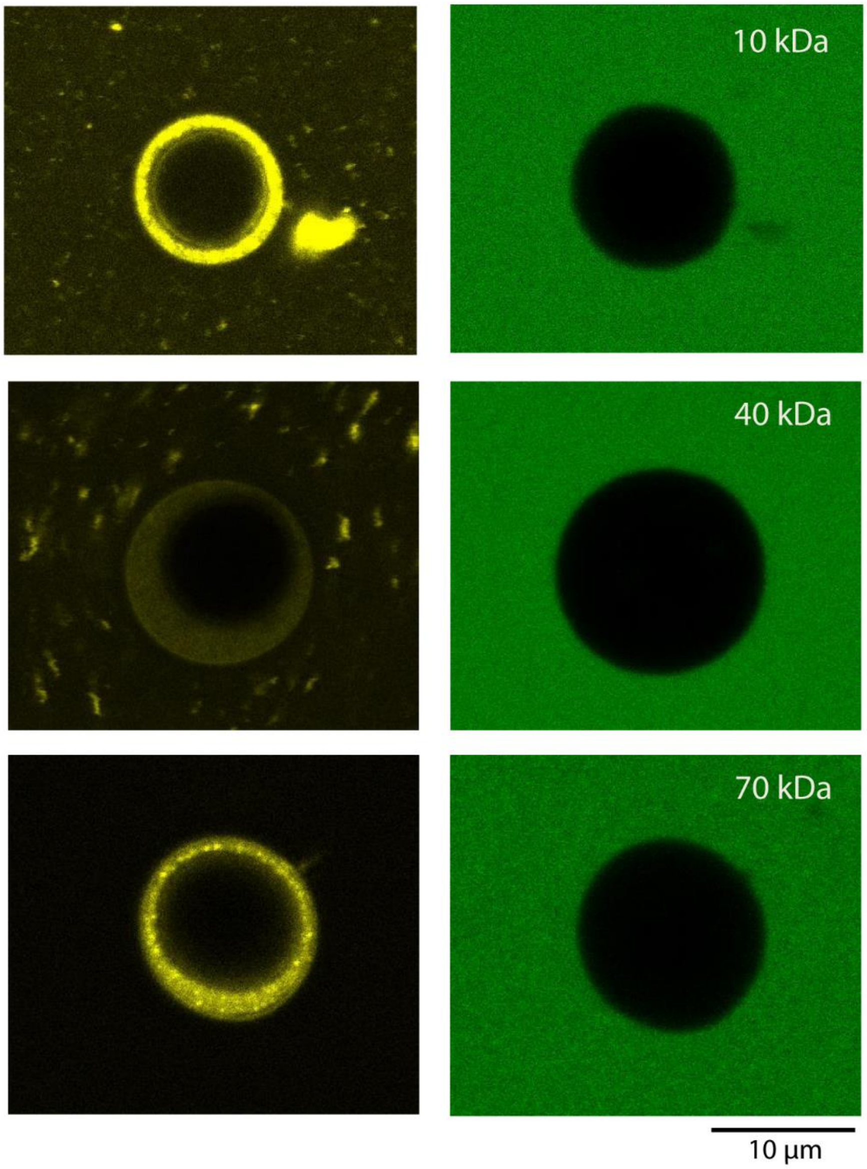

Supplementary Figure S3 CLSM images of 7-day stressed *Chlamydomonas reinhardtii* surrounded by various dextran molecules (10, 40 and 70 kDa). The cell wall is stained with Concanavalin A (yellow fluorescence) and the fluorescence of the dextran molecule is detected in green. All tested dextran molecules show no penetration in the 7-day stressed cell wall of *C. reinhardtii*.

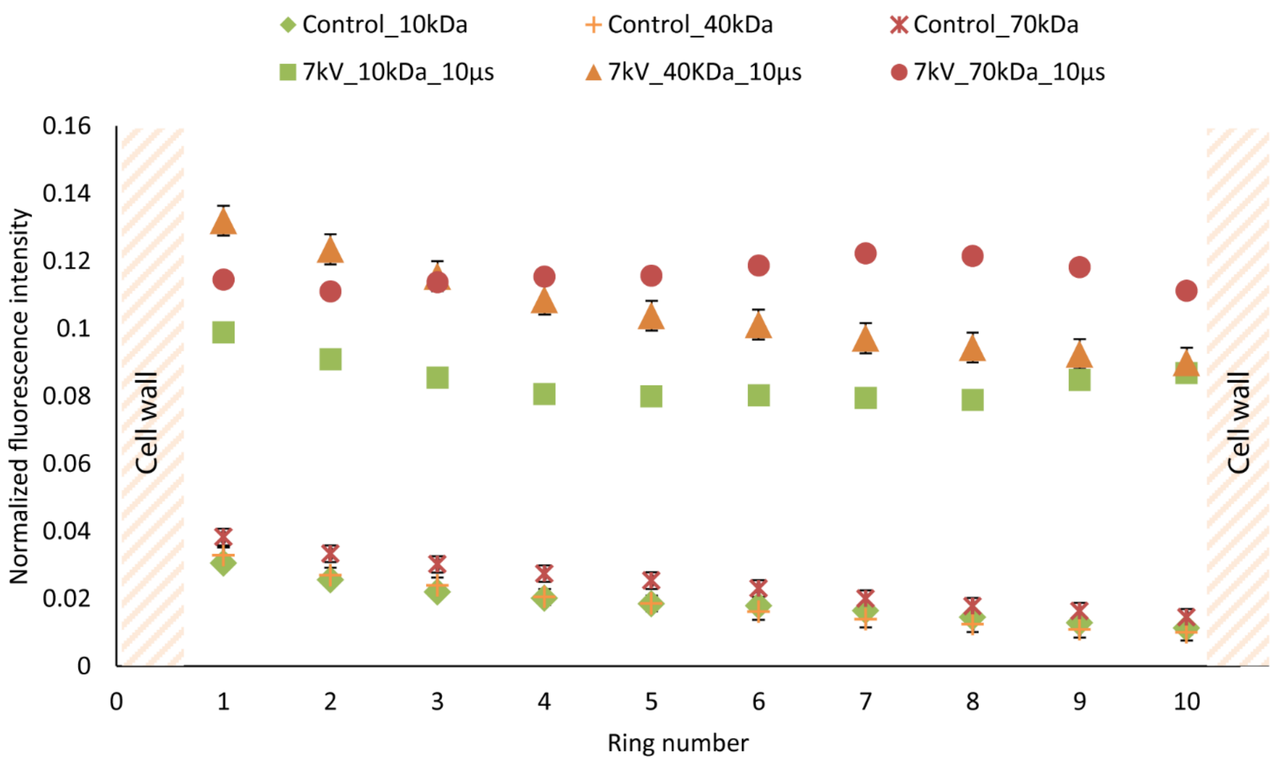

Supplementary Figure S4 Fluorescence intensity emitted by various dextran molecules (10 kDa, 40 kDa and 70 kDa) in algal cells previously submitted to irreversible electroporation (7 kV·cm^{-1}); ratio of the fluorescence intensity of given dextran emitted in each zone by the fluorescence intensity emitted in the medium. The other PEF parameters were kept constant: burst of 10 pulses, repetition frequency of 10 Hz and pulse duration of 10 µs. The control corresponds to 7-day stressed cells not submitted to any pretreatment.
